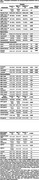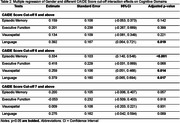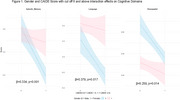# Impact of Cerebrovascular Disease on Gender‐Specific Cognitive and Biomarker Profiles: Insights from the Southeast Asian BIOCIS Cohort

**DOI:** 10.1002/alz70860_100577

**Published:** 2025-12-23

**Authors:** Pricilia Tanoto, Yi Jin Leow, Nagaendran Kandiah

**Affiliations:** ^1^ Dementia Research Centre (Singapore), Lee Kong Chian School of Medicine, Nanyang Technological University, Singapore, Singapore; ^2^ Lee Kong Chian School of Medicine, Nanyang Technological University, Singapore, Singapore; ^3^ Neuroscience and Mental Health Programme, Lee Kong Chian School of Medicine, Nanyang Technological University, Singapore, Singapore; ^4^ National Healthcare Group, Singapore, Singapore

## Abstract

**Background:**

Emerging evidence suggests that biological sex influences cognitive performance across various domains. However, data specific to Southeast Asian populations remain scarce. This study aims to delineate gender‐specific cognitive profiles and cerebrovascular risk among cognitively unimpaired individuals within the Southeast Asian BIOCIS cohort, thereby identifying potential disparities pertinent to this demographic.

**Method:**

The study included 714 cognitively unimpaired, age‐matched participants (357 males and 357 females) from the BIOCIS cohort. Each participant underwent a comprehensive neuropsychological assessment, which included the Montreal Cognitive Assessment (MoCA), the Visual Cognitive Assessment Test (VCAT), and tests evaluating episodic memory, executive function, processing speed, visuospatial abilities, and language. Demographic differences were analyzed using independent t‐tests and chi‐square tests. Gender differences in cognitive performance and interaction effects with cerebrovascular risk (CAIDE scores) were examined using multilinear regression models, adjusting for CAIDE scores.

**Result:**

The mean age of participants was 56.46±10.29 years, with an average education level of 15.53±3.22 years. Males had a significantly higher prevalence of a CAIDE score of 5 or above (*p* <0.001), hypertension (*p* = 0.001), and higher BMI (*p* <0.001), while females had a higher prevalence of hyperlipidaemia (*p* = 0.004). Neuropsychological assessments showed females scored higher in episodic memory and language (*p* <0.001), and males scored higher in visuospatial abilities (*p* <0.001). Multivariable regression, adjusted for CAIDE score, revealed significant gender differences in episodic memory (*p* = 0.007), executive function (*p* = 0.042), visuospatial (*p* < 0.001), and language (*p* <0.001). Gender differences varied with CAIDE score ≥6 (Table 2, Figure 1).

**Conclusion:**

This study identifies distinct gender‐specific cognitive, neuroimaging, and biomarker profiles in a Southeast Asian population. Females outperformed males in episodic memory and language, while males excelled in visuospatial abilities. Biomarker differences, such as elevated GFAP in females and higher *p*‐tau 181 in males, suggest underlying neurobiological mechanisms. Neuroimaging showed males having higher WMH volumes and females with greater gray matter volumes. Among those with a CAIDE score ≥6, females demonstrated smaller declines in episodic memory, language, and visuospatial abilities. These findings highlight the need for research to develop tailored assessments and interventions addressing gender‐specific cognitive and biological profiles.